# Peripheral Odontogenic Fibroma: A Rare Tumor mimicking a Gingival Reactive Lesion

**DOI:** 10.5005/jp-journals-10005-1416

**Published:** 2017-02-27

**Authors:** Komal Khot, Swati Deshmane, Kriti Bagri-Manjrekar, Paresh Khot

**Affiliations:** 1 Professor, Department of Oral Pathology and Microbiology, YMT Dental College & Research Institute, Navi Mumbai, Maharashtra, India; 2 Postgraduate Student, Department of Oral Pathology and Microbiology, YMT Dental College & Research Institute, Navi Mumbai, Maharashtra, India; 3 Reader, Department of Oral Pathology and Microbiology, YMT Dental College & Research Institute, Navi Mumbai, Maharashtra, India; 4 Private Practitioner, Dr. Khot’s Dental Clinic, Mumbai, Maharashtra, India

**Keywords:** Fibroma, Pediatric, Peripheral odontogenic fibroma, Peripheral odontogenic tumor.

## Abstract

Gingival growths are one of the most frequently encountered lesions in the oral cavity. A plethora of lesions can be seen having similar clinical presentation, making diagnosis a dilemma. Peripheral odontogenic tumors are rare neoplasms to occur on gingiva, the most common among them being the peripheral odontogenic fibroma (POdF). The POdF is a benign, slow-growing, exophytic lesion. Although considered to have a recurrence potential after excision, the actual recurrence rate is not known due to paucity of reported cases.

Presented here is a case of a rare neoplasm mimicking an inflammatory gingival lesion with review of the available literature.

**How to cite this article:**

Khot K, Deshmane S, Bagri-Manjrekar K, Khot P. Peripheral Odontogenic Fibroma: A Rare Tumor mimicking a Gingival Reactive Lesion. Int J Clin Pediatr Dent 2017;10(1):103-106.

## INTRODUCTION

Oral mucosa is constantly exposed to varied stimuli and, therefore, manifests a spectrum of diseases that range from developmental, reactive, and inflammatory to neoplastic.^1^ Growths on gingiva are common and may be due to underlying systemic disease, drug-induced stimulus, or local iatrogenic factors.^2^ Chronic trauma can induce inflammation, which produces granulation tissue that later proliferates and manifests as a tumor-like lesion called reactive hyperplasia.^3^

Cook regarded all pedunculated swellings from a mucosal surface as polyps, and these mostly occurred in the line of occlusion.^4^ All pedunculated and sessile lesions in the gingiva were designated as “epulis,” which commonly occurred in the maxillary anterior region. These appear on the interdental papilla as a result of local irritation from calculus, caries, or irregular restoration margins. Focal fibrous hyperplasia is the commonest epulis occurring over a wide age range (9-80 years), with peak incidence in the 3rd to 4th decade.

Kfir et al^5^ have classified gingival lesions into pyogenic granuloma (PG), peripheral giant cell granuloma (PGCG), fibrous hyperplasia, and peripheral fibroma with calcification.

Currently, localized reactive hyperplastic lesions of the gingiva have been classified as focal fibrous hyperplasia, PG, peripheral ossifying fibroma (POF), and PGCG.

Despite similarities, all reactive gingival lesions show some differences in sex, type, location, duration, and histological features. Although benign in nature, they do have a tendency toward recurrence with incomplete removal of the lesion or the local irritants involved at the site.^6^ The treatment in each case is surgical excision; however, different treatment modalities may offer better outcomes with less frequency of recurrence.

## CASE REPORT

**Fig. 1:**
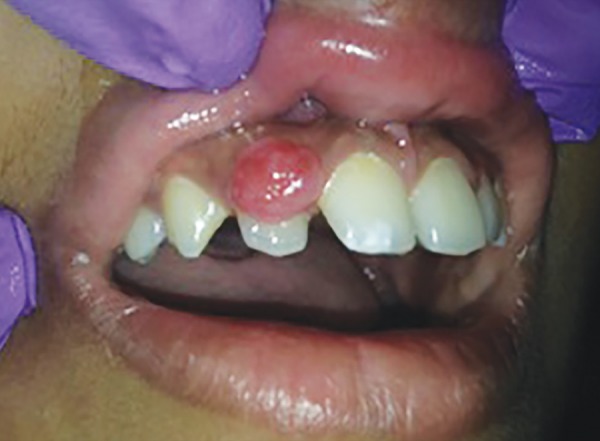
Preoperative clinical presentation of the lesion

A 14-year-old boy reported to the Department of Oral and Maxillofacial Pathology with an asymptomatic firm and sessile gingival mass in between 12 and 13 (Fig. 1). Swelling had gradually increased over a period of 2 years to the present size. No discharge or redness was observed, and mucosa was normal in appearance. The patient’s physical status was good and investigation of his medical history was not relevant. A provisional diagnosis of fibroma was made with a differential diagnosis of PGCG and POF. There were no significant radiographic findings on periapical view, performed with a small standard film to verify possible calcification or osseous involvement. An informed consent was obtained from the parents and patient. The surgical modality included excision of the lesion under local anesthesia following standard aseptic precautions. The excised tissue was subjected to histopathological examination.

**Fig. 2:**
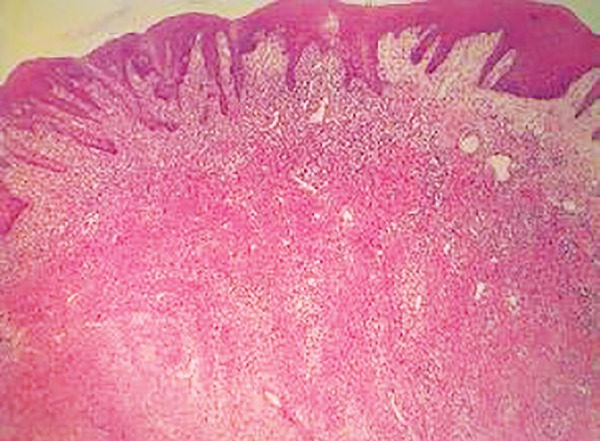
Proliferating keratinizing stratified squamous epithelium into underlying connective tissue (4x)

**Fig. 3:**
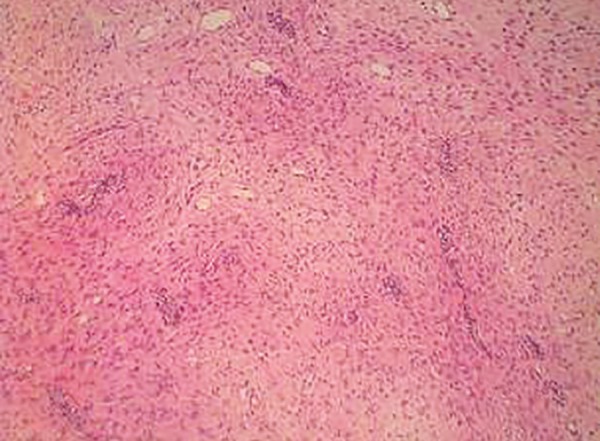
Islands of odontogenic epithelium within a cellular fibrous stroma (10x)

**Fig. 4:**
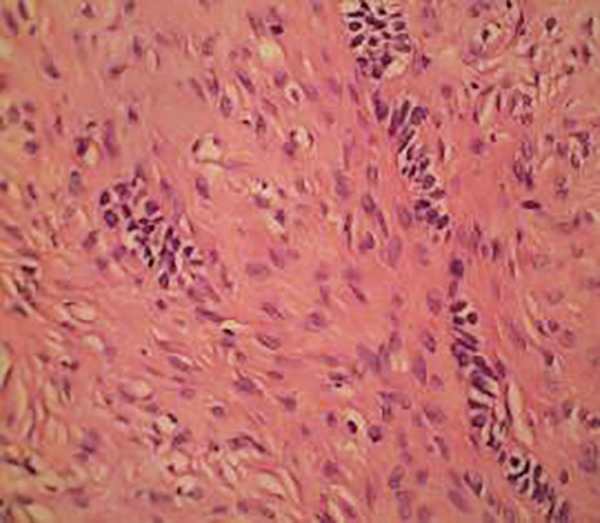
Islands of odontogenic epithelium within a cellular fibrous stroma (40x)

**Fig. 5:**
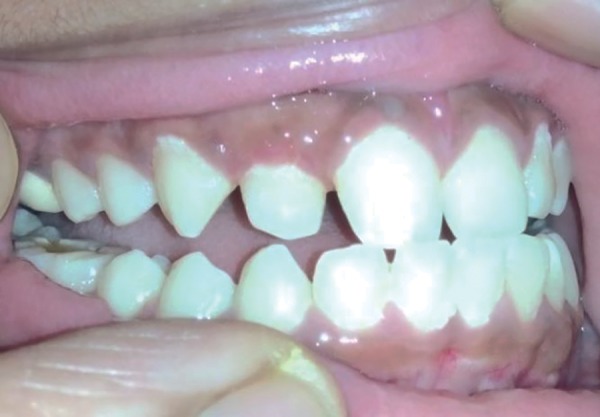
Postoperative view

The hematoxylin and eosin-stained sections revealed a lesion consisting of fibrocellular connective tissue with multiple small islands and strands of odontogenic epithelium resembling remnants of the dental lamina (Figs 2 to 4). The connective tissue was overlined by proliferating parakeratinized stratified squamous epithelium. Histological diagnosis of peripheral odontogenic fibroma (POdF) was established. The patient was followed up for 6 months and showed no evidence of any recurrence (Fig. 5).

## DISCUSSION

According to the World Health Organization, the odon-togenic fibroma is a relatively rare benign odontogenic neoplasm.^7^ In the past, some authors had designated clinically and histopathologically lesions similar to POdF as odontogenic gingival epithelial hamartoma/peripheral fibroblastic dentinoma, odontogenic epithelial hamartoma.^8^ The reason for it being referred to as odontogenic gingival epithelial hamartoma in the past is a reflection of the authors placing more emphasis on the epithelial component rather than the fibroblastic component. It does not appear to be a hamartoma because hamartomas are developmental in nature, while lesions reported as hamartomas in this particular study by Baden et al^9^ appeared only in 6th and 7th decades of life rather than during the development of dentition, which means that they were not hamartomas.

The first detailed clinicopathologic study of POdF was published by Daley and Wysocki,^10^ who reported POdF to be the third most common odontogenic tumor and the most common peripheral odontogenic tumor (POT).

**Table 1:** **Table 1:** Age, gender, and location of POdFs in pediatric populations described in the literature

*Case no*		*Study*		*Pediatric cases*		*Gender*		*Location*		*Age*	
1		Buchner et al		1		M		Maxilla		2	
2		Weber et al		1		F		Maxilla		8	
3		Daley et al		1		M		Mandible		19	
4				1		M		Mandible		13	
5		Buchner et al		1		F		Mandible		18	
6				1		F		Maxilla		16	
7				1		M		Mandible		18	
8				1		F		Mandible		16	
9				1		M		Mandible		18	
10		Alaeddini et al		1		M		Mandible		17	

A recent study of a series of 17 cases reports the peak incidence of POdF to be in the 2nd and 4th decades. Literature and published case reports indicate that POdF is very rare in children. Table 1 shows details of published cases of this tumor in pediatric patients. Ritwik and Brannon in their study found the site predilection of POdF to be in the incisor-cuspid region (74.5%). This site predilection is similar to the 60% of POdF lesions found in the incisor-cuspid region in the 59 cases reported by Daley and Wysocki.^10^ A slight preponderance for the female gender was observed.

Histogenesis of the POdF has not been established, but derivation from each tissue component resident to the site of occurrence has been speculated, namely from dental lamina rests, ectomesenchyme, surface epithelium, and the periodontal ligament. Several studies have suggested that the rests of dental lamina that persist in the gingiva following disintegration of the dental lamina may be the embryologic source of the POdF.^7^ Farman^11^ suggested that the ectomesenchyme in the gingiva may induce secondary proliferation of dental lamina remnants and also of the basal layer of the gingival epithelium.

In a case series, Ritwik and Brannon^7^ found characteristic surface epithelium features, such as slender, deep penetrating rete ridges seen in 147 (97%) cases. Budding or bud-like extensions from the basal layer of the surface epithelium were seen in 86 (57%) lesions. The prominent histopathologic findings were the surface epithelium activity, the presence of odontogenic epithelial rests, and the connective tissue stroma, which ranged from predominantly fibrous to predominantly myxoid. All of these components which are essential to the microscopic diagnosis of POdF were seen in our case.

The recurrence rate of POdF has been reported to be from very low to as high as 38.9%.^12^ Ritwik and Brannon,^7^ in their case series, found that lesions that had calcifications in opposition to odontogenic rests were less likely to recur. On the contrary, lesions without calcifications, but with active surface epithelium in the form of budding of the basal cell layer of the rete ridges were associated with significantly higher recurrence. The recurrence of POdF is associated with two histologic variables: (1) Budding of the basal cells is associated with a significantly higher recurrence of this lesion. (2) The presence of calcifications in opposition to odontogenic epithelial rests is associated with a significantly lower recurrence.

The differential diagnosis includes inflammatory lesions, such as fibrous hyperplasia, fibroma, giant cell fibroma, POF, POTs, PGCG, and PG.

The PG is an inflammatory reactive lesion, which occurs as an exophytic and erythematous mass with a sessile or pedunculated base.^13^ The PG is usually present in the maxillary gingiva, and the PG’s histological analysis shows a vascular proliferation similar to granulation tissue.

The POF is a reactive lesion characterized by a soft tissue growth nodule and is, probably, originated from the periodontal ligament fibers. It can be sessile or pedun-culated and its appearance may vary from pink to red (ulcerated) and from smooth to irregular surfaces. Mineralization focuses are commonly observed, presenting a cementum-like, bone or dystrophic calcification arrangement. Also, there are some histopathologic similarities involving POF and POdF that deserve attention by the pathologists. Both consist of fibroblastic proliferation and may share bone, cementum-like, or dystrophic calcifications, although Sciubba and Zola^8^ showed that the main difference between these two lesions is the presence of the odontogenic epithelium present, only, in POdF.

The PGCG can originate from the periosteum or periodontal membrane, which appears clinically as a red-purple nodule. It occurs mostly in patients between the 5th and 6th decades of life, while POdF is more restricted from the 2nd to 4th decades and usually present in the posterior area. The PGCG mass has characteristic hemorrhage, deposits of hemosiderin, ovoid spindle cells, and large number of multinucleate giant cells. Histologically, POdF may present as multinucleated giant cells, but these cells are present in a lower quantity than in PGCG. The presence of odontogenic epithelium and its collagenous stroma - richly composed by fibroblastic cells - with a fasciculate arrangement is essential for the diagnoses of POdF.

## CONCLUSION

The POdF is a rare odontogenic tumor, which is frequently misdiagnosed clinically as an inflammatory lesion. Although it shares many characteristics with other gingival lesions, there are pathognomonic features that can guide the clinical and the pathologist to arrive at an accurate diagnosis. The POdF exhibits a significant growth potential and recurrence. Therefore, it is important to maintain a close postoperative follow-up.

## References

[1] Ezirganl S, Taşdemir U, Göze F, Kara MI, Polat S, Muderris S (2014). Intraoral localized reactive hyperplastic lesions in Sivas.. ACU Saglik Bil Derg.

[2] Ramu S, Rodrigues C (2012). Reactive hyperplastic lesions of the gingiva: a retrospective study of 260 cases.. World J Dent.

[3] Cooke BE (1952). The fibrous epulis and fibro epithelial polyp: their histogenesis and natural history.. Br Dent J.

[4] Reddy V, Saxena S, Saxena S, Reddy M (2012). Reactive hyperplastic lesions of the oral cavity: a ten year observational study on North Indian Population.. J Clin Exp Dent.

[5] Kfir Y, Buchner A, Hansen LS (1980). Reactive lesions of the gingiva. A clinicopathological study of 741 cases.. J Periodontol.

[6] Kashyap B, Reddy PS, Nalini P (2012). Reactive lesions of oral cavity: a survey of 100 cases in Eluru, West Godavari district.. Contemp Clin Dent.

[7] Ritwik P, Brannon RB (2010). Peripheral odontogenic fibroma: a clini-copathologic study of 151 cases and review of the literature with special emphasis on recurrence.. Oral Surg Oral Med Oral Pathol Oral Radiol Endod.

[8] Sciubba JJ, Zola MB (1978). Odontogenic epithelial hamartoma.. Oral Surg Oral Med Oral Pathol.

[9] Baden E, Moskow BS, Moskow R (1968). Odontogenic gingival epithelial hamartoma.. J Oral Surg.

[10] Daley TD, Wysocki GP (1994). Peripheral odontogenic fibroma.. Oral Surg Oral Med Oral Pathol.

[11] Farman AG (1975). The peripheral odontogenic fibroma.. Oral Surg Oral Med Oral Pathol.

[12] Kamal R, Palaskar S, Shetty VP, Bhushan A (2008). Multifocal peripheral odontogenic fibroma.. J OralMaxillofac Pathol.

[13] de Freitas Silva BS, Yamamoto FP, Da Costa RMB, Cruz e Silva BT, de Carvalho WRS, Pontes HAR (2012). Peripheral odontogenic fibroma: case report of a rare tumor mimicking a gingival reactive lesion.. Rev Odontol UNESP.

